# Functions and underlying mechanisms of lncRNA HOTAIR in cancer chemotherapy resistance

**DOI:** 10.1038/s41420-022-01174-3

**Published:** 2022-09-13

**Authors:** Chunming Zhu, Xia Wang, Yuan Wang, Kefeng Wang

**Affiliations:** 1 Department of Family Medicine, Shengjing Hospital of China Medical University, Shenyang, 110004 China; 2 Department of Urology, Shengjing Hospital of China Medical University, Shenyang, 110004 China; 3 Department of General Surgery, Shengjing Hospital of China Medical University, Shenyang, 110004 China

**Keywords:** Long non-coding RNAs, Chemical genetics

## Abstract

Chemotherapy has been one of the most important treatments for advanced cancer in recent decades. Although the sensitivity rate of initial chemotherapy is high, patients with chemotherapy resistant tumors, experience tumor recurrence. In recent years, many studies have shown that homeobox transcript antisense intergenic RNA (HOTAIR) is involved in many pathological processes including carcinogenesis. The abnormal regulation of a variety of cell functions by HOTAIR, such as apoptosis, the cell cycle, epithelial-mesenchymal transition, autophagy, self-renewal, and metabolism, is associated with chemotherapy resistance. Therefore, there is an urgent need to understand the biology and mechanism underlying the role of HOTAIR in tumor behavior and its potential as a biomarker for predicting the effect of chemotherapy. In this manuscript, we review the mechanisms underlying HOTAIR-related drug resistance and discuss the limitations of current knowledge and propose potential future directions.

## Facts

- Cancer has become a major threat to human health, but there is no effective way to stop its progress.
- Chemotherapy plays an important role in controlling tumor growth, but chemotherapy resistance is still a difficult problem to be solved.
- HOTAIR has been shown to be abnormally expressed in a variety of cancers and is associated with poor prognosis.
- With the emergence of tumor resistance, HOTAIR has also been confirmed to be involved in chemotherapy resistance of tumors.

## Open Questions

- What is the specific mechanism of HOTAIR’s involvement in tumor chemotherapy resistance?
- How to reduce drug resistance by inhibiting the expression of HOTAIR in clinic?

## Introduction

Long non-coding RNAs (lncRNAs) are non-coding RNAs with a length of over 200 nucleotides [1]. LncRNAs are mainly transcribed by RNA polymerase II from different regions of the entire genome. Growing evidence shows that lncRNAs are involved in a variety of carcinogenic processes, such as tumor proliferation, invasion, and metastasis [2–4]. The role of lncRNAs in tumorigenesis and tumor progression has been widely investigated. However, studies of the role of lncRNAs in cancer resistance to chemotherapy are still at a nascent stage [5].

**Fig. 1:**
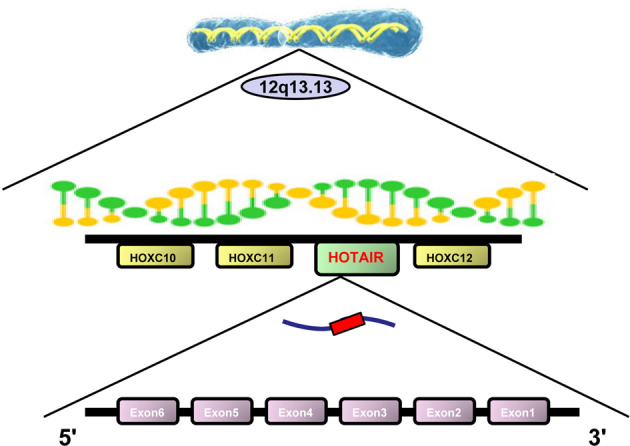
Schematic location of HOTAIR. HOTAIR is transcribed from the antisense strand of the HOXC gene, which is located between HOXC11 and HOXC12 on chromosome 12. HOTAIR is a lncRNA, containing 6 exons and 2158 nucleotides.

Homeobox transcript antisense intergenic RNA (HOTAIR) is an important polyadenylated and spliced lncRNA, containing 6 exons and 2158 nucleotides [6]. HOTAIR is transcribed from the antisense strand of the *HOXC* gene, which is located between *HOXC11* and *HOXC12* on chromosome 12q13.13 (Fig. 1) [7]. Abnormal overexpression of HOTAIR in cancer was first identified in breast cancer (BC) and has been associated with metastasis and poor survival [8]. Elevated expression of HOTAIR was found to induce genome-wide retargeting of polycomb repressive complex 2 (PRC2), leading to increase metastasis and invasion in BC cells [8]. Since then, HOTAIR has been attracting attention in the field of cancer.

**Fig. 2:**
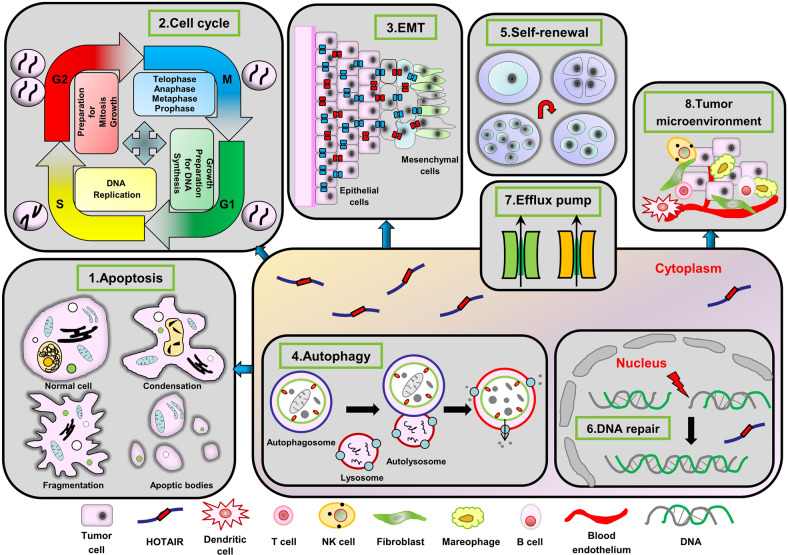
Roles of HOTAIR in mechanisms mediating chemotherapy resistance of cancers. This schematic shows the mechanism of HOTAIR-mediated drug resistance in cancer cells. (1) inhibition of cell apoptosis, (2) avoidance of cell cycle checkpoints, (3) enhancement of EMT, (4) reconstitution of cell autophagy, (5) enhancement of cell self-renewal, (6) remodeling DNA repair, (7) alteration of efflux pump, (8) and modulation of tumor microenvironment.

Chemotherapy plays an important role in controlling tumor growth and can increase the survival time of patients [9]. Several studies have shown that chemotherapy can significantly reduce the mortality rate of patients with advanced tumors and those who cannot undergo surgery [10, 11]. Despite the effectiveness of chemotherapy, the acquired chemotherapeutic resistance is a huge challenge to cancer treatment. Chemotherapy resistance can lead to tumor recurrence and increase patient mortality. The mechanisms underlying the development of resistance in cancer cells involve: (1) inhibition of cell apoptosis and protection of damaged cells, (2) avoidance of cell cycle checkpoints, (3) enhancement of epithelial-mesenchymal transition (EMT), (4) reconsitution of the cell autophagy system, (5) enhancement of self-renewal ability, (6) remodeling the repair ability of DNA, (7) alteration of drug metabolism and transport, which affects the pharmacokinetics in cancer cells, (8) and modulation of the tumor microenvironment (TME) (Fig. 2) [12–14]. In this review, we discuss the research progress on the relationship between HOTAIR and cancer chemotherapy resistance.

## HOTAIR biogenesis and general functions

**Fig. 3:**
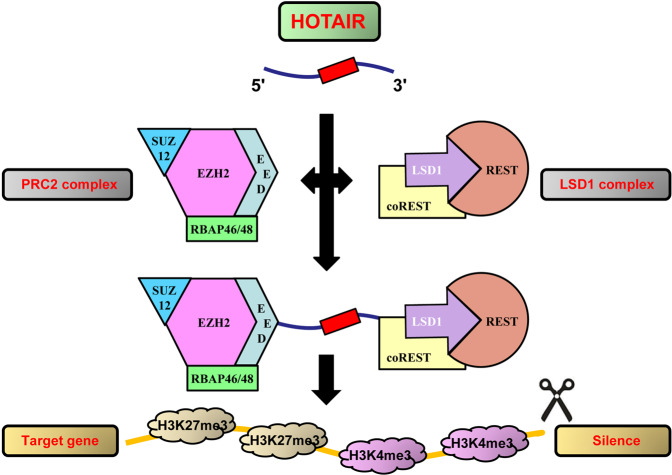
Functions and molecular mechanisms of HOTAIR. HOTAIR can recruit PRC2 and LSD1 complexes and act as a bridge. HOTAIR guides these complexes to the target genes, thereby regulating the trimethylation of H3K27 and demethylation of H3K4.

HOTAIR, first identified by Rinn et al. in 2007 [6], can inhibit the transcription of 40 kb at the *HOXD* site. HOTAIR is a key regulator of transcriptional silencing and chromatin remodeling. HOTAIR interacts with some vital epigenetic regulators, such as PRC2 and lysine-specific histone demethylase 1 A (LSD1) to regulate gene silencing [15, 16]. The PRC2 complex contains four main subunits, namely, SUZ12, EED, EZH2, and RBAP46/48, and interacts with a fragment at the 5ʹ end of HOTAIR [17]. Silencing of HOTAIR could activate the transcriptional repression of the HOXD site on chromosome 2 by reducing the tri-methylation of H3 lysine 27 trimethylation (H3K27me3), which is the feature of gene silencing modulated by EZH2, a member of the PRC2 family [18]. Another mechanism underlying the mode of interaction of HOTAIR with PRC2 has been proposed, according to which PRC2 interacts with short repeats of successive guanines in HOTAIR rather than with specific domains (Fig. 3) [19]. Recent studies revealed that RNA G-quadruplexes (G4s) at the 5ʹ end of HOTAIR mediated the reciprocal interaction with PRC2 [20]. RNA G4s are made by stacking two or more connected square planes of four guanines and are involved in many important cellular processes and the pathogenesis of various diseases, such as cancer [21]. Besides PRC2, the LSD1 complex is another important partner of HOTAIR, which interacts with a fragment at the 3ʹ end of HOTAIR (Fig. 3) [16]. The LSD1 complex consists of LSD1, REST, and CoREST, which inhibit gene expression by decreasing the tri-methylation of histone H3 Lys 4 (H3K4me3). In addition, recent studies have shown that HOTAIR influences miRNA-mediated inhibition of target gene expression through competitive binding with miRNA [22, 23]. Furthermore, HOTAIR alters gene expression by pairing with translation factors or ribosomal bases to control translation. In conclusion, HOTAIR functions as a scaffold to assemble the inhibitory complex composed of PRC2 and LSD1, thereby silencing its target genes through H3K27me3 (PRC2 activity) and H3K4me3 (LSD1 activity), respectively.

HOTAIR is found not only in humans, but also in mice and rats. However, it does not exist in non-mammalian vertebrates [24]. The sequence similarity between mouse HOTAIR and human HOTAIR is about 58%, while it is about 50% in rat HOTAIR [25, 26]. Therefore, HOTAIR in mammalian vertebrates is conservative. The human HOTAIR gene has 6 exons, whereas the rat and mouse HOTAIR gene has only 5 exons because exon 2 is not present [26]. There are two domains in exon 6 of rat and mouse HOTAIR, called domains A and B. Domain B is responsible for the interaction between HOTAIR and plenty of proteins [26]. However, the physiological function of domain A remains unclear.

HOTAIR is a vital modulator of chromatin dynamics. It acts on multiple genetic sites and modulates their expression [27]. HOTAIR interacts with RNA, DNA, and proteins to participate in a variety of cellular functions. During mammalian embryogenesis, HOTAIR is expressed in the forelimb and wrist, hindlimb bud and posterior trunk, and genital bud and tail [28]. In humans, HOTAIR is highly concentrated in the skin and reproductive system, mainly involving endometrium, prostate and testis [28]. HOTAIR can down-regulate osteogenic genes BMP2 and ALPL, indicating that HOTAIR has a negative regulatory role in the process of osteogenesis [29]. HOTAIR is involved in cell senescence by interacting with E3 ubiquitin ligases [30]. HOTAIR also participates in cell proliferation by regulating the expression of cell cycle-related proteins and kinases [28].

## Mechanisms mediating HOTAIR-related chemotherapy resistance in cancer

### HOTAIR regulates cell apoptosis and cell cycle

The ability of cancer cells to respond to chemotherapy drugs is thought to be due to their ability to undergo apoptosis. Drug-induced apoptosis is not only modulated by an upregulation of pro-apoptotic factors but also regulated by cell survival factors [31].

Recent studies revealed that HOTAIR is involved in the modulation of the apoptotic pathway in cancer cells, which may be related to chemotherapy resistance (Table 1). For example, a study showed that HOTAIR suppression promoted apoptosis and doxorubicin sensitivity in acute myeloid leukemia (AML). Furthermore, an investigation of the mechanism indicated that HOTAIR conferred multidrug resistance (MDR) to AML cells by modulating the expression of the AKT/Notch1 and P21 signaling pathways [32]. Another research group reported that HOTAIR inhibited PTEN expression in a manner dependent on DNMT3b upregulation, which resulted in doxorubicin resistance in AML [33]. In addition, downregulation of HOTAIR played a vital role in enhancing the acquired resistance to imatinib via PI3K/AKT signaling in chronic myelogenous leukemia (CML) [34]. Furthermore, the PI3K/AKT signaling pathway was found to be involved in HOTAIR-mediated cell apoptosis and chemotherapy resistance in BC and gastric cancer (GC) [35, 36]. Similar results have been widely reported in other digestive system tumors. The study by Zhang et al. [37] revealed that targeting HOTAIR sensitized esophageal cancer cells to 5-fluorouracil (5-FU) chemotherapy through the downregulation of methylenetetrahydrofolate reductase expression. In hepatocellular carcinoma (HCC), downregulation of HOTAIR weakened Taxol resistance through the Wnt/β-catenin and Akt phosphorylation pathways via antagonizing miR-34a [38]. In pancreatic cancer, high HOTAIR expression expedited cell resistance to TNF-related apoptosis-inducing ligand (TRAIL)-induced apoptosis through modulation of TRAIL receptor death receptor 5 expression [39]. Recently, it was reported that HOTAIR expression in non-small cell lung cancer (NSCLC) was dramatically higher than that in adjacent tissues and that high HOTAIR expression was associated with shortened overall patient survival. Small interfering RNA targeting HOTAIR (si-HOTAIR) reversed the sensitivity of NSCLC cells to cisplatin (DDP) [40]. Another study demonstrated that H3K27me3 affected cell apoptosis and HOXA1 methylation through HOTAIR modulation, indicating that targeting H3K27me3 could be an effective strategy against lung cancer chemotherapy resistance [41]. In osteosarcoma, HOTAIR increased DDP resistance by affecting cell apoptosis. Functional assays demonstrated that suppression of HOTAIR decreased DDP resistance and promoted cell apoptosis via miR-106a-5p/STAT3 signaling [42]. In endometrial cancer, HOTAIR repressed progesterone receptor B expression and mediated progesterone sensitivity. Hence, si-HOTAIR could be a potential therapeutic target for overcoming progesterone resistance [43]. In glioblastoma (GBM), the depletion of HOTAIR restrained HK2 expression by regulating miR-125, which facilitated cell apoptosis and elevated temozolomide (TMZ) sensitivity [44].

**Table 1:** HOTAIR inhibits cell apoptosis.

Cancer type	Expression	Related drugs	Target	Related genes or pathway	References
Leukemia	upregulation	doxorubicin	/	AKT/Notch1 and P21	[32]
Leukemia	upregulation	doxorubicin	/	PTEN	[33]
Leukemia	upregulation	imatinib	/	PI3K/Akt	[34]
Breast cancer	upregulation	doxorubicin	/	PI3K/Akt/mTOR	[35]
Gastric cancer	upregulation	cisplatin	miR-34a	PI3K/Akt and Wnt/β-catenin	[36]
Esophageal cancer	upregulation	5-fluorouracil	/	MTHFR	[37]
Hepatocellular cancer	upregulation	taxol	miR-34a	Akt and Wnt/β-catenin	[38]
Pancreatic cancer	upregulation	/	/	EZH2/DR5	[39]
Lung cancer	upregulation	cisplatin	/	Wnt	[40]
Lung cancer	upregulation	/	/	HOXA1	[41]
Osteosarcoma	upregulation	cisplatin	miR-106a-5p	STAT3	[42]
Endometrial cancer	upregulation	progesterone	/	PRB	[43]
Glioblastoma	upregulation	temozolomide	miR-125	HK2	[44]

In summary, many genes and proteins involved in the inhibition or induction of apoptosis, such as p53, inhibitor of apoptosis family (including X-linked inhibitor of apoptosis protein, neuronal apoptosis inhibitor protein, livin, survivin, and human inhibitor of apoptosis protein), Fas, TNF receptor, TRAIL receptor, PI3K/AKT, FAK, prohibitin, BAK, BID, BAD, BCL, and caspases, are abnormally regulated in various tumors [31, 45–49]. HOTAIR depletion could be an effective way to promote cell apoptosis and increase chemotherapeutic sensitivity in multiple cancers.

The cell cycle is regulated by various molecules, including cyclins, cyclin-dependent kinases, cyclin-dependent kinase inhibitors, and cell cycle checkpoints. Defects of cell cycle checkpoint kinases and promotion of cell cycle progression are associated with changes in the response of cancer cells to chemotherapy.

A large number of studies have shown the vital role of HOTAIR in modulating the cell cycle and driving chemotherapy resistance (Table 2). For example, the HOTAIR expression level was upregulated in DDP-resistant adenocarcinoma cells compared to that in A549 cells. si-HOTAIR-mediated recession of chemotherapy resistance was associated with the induction of G0/G1 arrest via modulation of p21 expression [50]. Similarly, the study by Fang et al. [51] revealed that suppression of HOTAIR expression prolonged the S phase and increased cell sensitivity to DDP, adriamycin (ADM), and etoposide (VP-16) by modulating HOXA1 methylation in small cell lung cancer (SCLC). The collection and analysis of bioinformatic data has attracted increasing attention. Liu et al. [52] proposed that HOTAIR was related to cell cycle pathways using Kyoto Encyclopedia of Genes and Genomes analysis of The Cancer Genome Atlas public database. The underlying molecular mechanisms indicated that HOTAIR heightened cell resistance to gefitinib by accelerating the progression of the cell cycle from the G1 to S phase by modulating the Rb-E2F pathway. In another study, a traditional Chinese medicine formula Xiaoji decoction (XJD) was demonstrated to suppress cell proliferation and induce cell arrest at the G2/M phase in NSCLC. Mechanism analysis showed that XJD reinforced the effect of the antineoplastic drug gefitinib through downregulation of EP4 induced by HOTAIR silencing [53]. In GC, upregulation of HOTAIR facilitated G1 to S phase transition and led to DDP resistance; HOTAIR performed its function by sponging miR-126 to promote PI3K/AKT/MRP1 signaling [54]. Another study showed that abnormally overexpressed HOTAIR stimulated cell proliferation and cell cycle transition to the S phase. Upregulation of HOTAIR increased doxorubicin and paclitaxel (PTX) resistance by suppressing the miR-217/GPC5/PTPN14 pathway [55]. In ovarian cancer (OC), HOTAIR enhanced PTX resistance through upregulation of CHEK1 expression. In contrast, si-HOTAIR, which could be used as a therapeutic target for OC, accelerated cell cycle arrest at the G2/M phase and boosted cell sensitivity to PTX [56]. The study by Xiang et al. [57] demonstrated that polyphyllin I (PPI), a main component extracted from *Rhizoma Paridis* saponins, played an anticancer role in prostate cancer. Further investigation into the mechanisms indicated that PPI suppressed cell growth at the S phase through downregulation of the expression of HOTAIR and its downstream targets DNMT1 and EZH2. In multiple myeloma (MM), downregulation of HOTAIR inhibited cell viability by inducing cell cycle arrest at the G0/G1 phase. Functional assays validated that HOTAIR enhanced cell activity and chemotherapy resistance to dexamethasone (DEX) via the JAK2/STAT3 pathway in MM [58].

**Table 2:** HOTAIR participates in cell cycle regulation.

Cancer type	Expression	Related drugs	Target	Related genes or pathway	References
Lung cancer	upregulation	cisplatin	/	P21	[50]
Lung cancer	upregulation	cisplatin, doxorubicin, and etoposide	/	HOXA1	[51]
Lung cancer	upregulation	gefitinib	/	Rb-E2F	[52]
Lung cancer	upregulation	gefitinib	/	EP4	[53]
Gastric cancer	upregulation	cisplatin	miR-126	PI3K/AKT/MRP1	[54]
Gastric cancer	upregulation	doxorubicin and paclitaxel	miR-217	GPC5/PTPN14	[55]
Ovarian cancer	upregulation	paclitaxel	/	CHEK1	[56]
Prostate cancer	upregulation	/	/	DNMT1 and EZH2	[57]
Myeloma	upregulation	dexamethasone	/	JAK2/STAT3	[58]

Taken together, the above data show that si-HOTAIR induces cell cycle arrest and decreases chemotherapy resistance in many cancers. Two important apical kinases, ataxia telangiectasia and Rad3-related and ataxia telangiectasia-mutated, are involved in the regulation of the relevant signal pathway through downstream signal relay proteins and effector proteins, such as CHK1, CHK2, MDM2, P53, BRCA1, PIK3, and CDC25 [59, 60]. Thus, it could be considered as a diagnostic biomarker and therapeutic target in cancer.

### HOTAIR enhances EMT

EMT refers to the transformation of epithelial cells into cells with mesenchymal phenotypes. EMT plays important roles in embryonic development, chronic inflammation, tissue remodeling, organ fibrosis, as well as cancer metastasis [61].

Several studies indicate that HOTAIR takes part in the regulation of EMT in cancer cells, affecting chemotherapy resistance (Table 3). It has been reported that the HOTAIR expression level was associated with chemotherapy resistance to epidermal growth factor receptor tyrosine kinase inhibitors through activating EMT in NSCLC. Upregulation of HOTAIR retarded gefitinib sensitivity in NSCLC cells, which served as a predictive biomarker for gefitinib resistance [62]. Another group reported that sorafenib resistance was accelerated in cells with abnormally overexpressed HOTAIR in HCC. An in-depth molecular mechanism analysis showed that si-HOTAIR enhanced sorafenib sensitivity by decreasing the Vimentin level and increasing the E-cadherin level by upregulating miR-217, which indicated that EMT may participate in HOTAIR-induced chemotherapy resistance [63]. The study by Jia et al. [64] showed that HOTAIR overexpression expedited the EMT process by regulating the miR-17-5p/PTEN pathway in GC. Therefore, anti-HOTAIR treatment boosted the sensitivity to chemotherapeutic drugs. In another study, suppression of HOTAIR expression influenced the expression of EMT-related genes in BC. HOTAIR downregulation using siRNA weakened trastuzumab resistance through activating the MEK/MAPK and PI3K/AKT/mTOR pathways [65]. In prostate cancer, propofol strengthened PTX sensitivity by regulating HOTAIR-mediated EMT [66]. In GBM, HOTAIR knockdown suppressed tumor growth, migration, and invasion and EMT, whereas exosomal HOTAIR accelerated TMZ resistance by the miR-519a-3p/RRM1 pathway [67].

**Table 3:** HOTAIR enhances EMT.

Cancer type	Expression	Related drugs	Target	Related genes or pathway	References
Lung cancer	upregulation	gefitinib	/	Rb-E2F	[62]
Hepatocellular cancer	upregulation	sorafenib	miR-217	Vimentin and E-cadherin	[63]
Gastric cancer	upregulation	cisplatin, adriamycin, mitomycin, and 5-fluoroura	miR-17-5p	PTEN	[64]
Breast cancer	upregulation	trastuzumab	/	MEK/MAPK and PI3K/AKT/mTOR	[65]
Prostate cancer	upregulation	paclitaxel	/	Bcl-2 and Bax	[66]
Glioblastoma	upregulation	temozolomide	miR-519a-3p	RRM1	[67]

Overall, emerging evidence shows that this process is driven by some EMT-inducing transcription factors (EMT-TFs), such as SNAI1/2, ZEB1/2, and TWIST1/2 [68]. EMT-TFs have been found to change chemotherapy resistance through several molecular mechanisms [69]. These data support the conclusion that HOTAIR enhances EMT and decreases chemotherapeutic sensitivity in various cancers.

### HOTAIR enhances autophagy

Autophagy is the process in which autophagosomes, consisting of cytoplasmic proteins or organelles enclosed into vesicles, fuse with lysosomes to form autophagy lysosomes, delivering their contents for degradation, to realize the metabolic needs of cells and the renewal of some organelles [70]. In cancer, autophagy is a double-edged sword, depending on the cellular environment and the characteristics of the tumors, including tumor stage, grade, type, and genetic relationships [71].

Recently, a series of studies revealed that HOTAIR plays a vital role in autophagy-mediated chemotherapeutic sensitivity in human cancers (Table 4). In NSCLC, HOTAIR silencing attenuated drug resistance to crizotinib through suppressing autophagy by inhibiting the ULK1 pathway [72]. Soon after, a study reported that HOTAIR knockdown inhibited autophagy by downregulating autophagy-related genes and increased drug sensitivity to DDP in oral squamous cell carcinoma [73]. The study by Zhang et al. [74] demonstrated that HOTAIR targeted miR-130a, the ATG2B inhibitor, to promote drug resistance to imatinib by elevating autophagy levels in gastrointestinal stromal tumors. Similar results indicated that HOTAIR modulated cisplatin resistance through the modulation of autophagy by affecting MDR, Beclin-1, and P-glycoprotein (P-gp) expression in endometrial cancer [75]. Another research group found that HOTAIR was overexpressed in OC and that ATG7 was the downstream gene of HOTAIR. Downregulation of HOTAIR enhanced DDP sensitivity by suppressing DDP-induced cell autophagy [76]. In renal cancer, HOTAIR targeted miR-17-5p to stimulate Beclin1-mediated autophagy, thereby increasing sunitinib resistance [77].

**Table 4:** HOTAIR enhances autophagy.

Cancer type	Expression	Related drugs	Target	Related genes or pathway	References
Lung cancer	upregulation	crizotinib	/	ULK1	[72]
Oral cavity carcinoma	upregulation	cisplatin	/	Autophagy-related gene	[73]
Gastrointestinal stromal tumor	upregulation	imatinib	miR-130a	ATG2B	[74]
Endometrial cancer	upregulation	cisplatin	/	MDR, Beclin-1, and P-gp	[75]
Ovarian cancer	upregulation	cisplatin	/	ATG7	[76]
Renal cancer	upregulation	sunitinib	miR-17-5p	Beclin1	[77]

Taken together, under normal or mild stress conditions, mild autophagy is conducive to cell survival, whereas excessive autophagy results in cell death [78]. The process of autophagy involves multiple steps, each of which is regulated by a set of core autophagy related proteins and transcription factors [79]. Therefore, the regulation of chemotherapeutic sensitivity by intervening with the regulatory factors involved in the autophagy process has become a promising new approach for cancer treatment. These data confirm that HOTAIR could enhance autophagy and decrease chemotherapeutic sensitivity in human tumors, providing the foundation for novel and promising antitumor therapy approaches.

### HOTAIR enhances the self-renewal ability of cancer stem cells

Cancer stem cells (CSCs) are cells with self-renewal abilities that lead to tumorigenesis [80, 81]. The abnormal self-renewal mechanism of CSCs leads to tumor cell proliferation and tumor tissue enlargement.

Recent studies have shown that lncRNAs regulate the stem cell properties of cancer cells (Table 5). Liu et al. [82] found that HOTAIR upregulation enhanced NSCLC cell resistance to DDP. Overexpression of HOTAIR increased the expression of CSC-related biomarkers and was correlated with Klf4 expression, which could be used as a promising therapeutic target. A year later, another group found that gemcitabine may induce HOTAIR as a tumor promoter. HOTAIR promotes PACN-1 CSC self-renewal ability, growth, and migration by inhibiting the chemosensitivity of PACN-1 CSCs, supporting its potential as a new therapeutic target for pancreatic cancer [83]. In BC, HOTAIR was found to be a vital regulator of the self-renewal ability of CSCs, which occurred partly through modulation of the miR-34a/Sox2/p53 signal pathway [84]. In prostate cancer, HOTAIR elevated the quantity of CSCs by activating the STAT3 signal pathway. Investigation of the mechanisms showed that HOTAIR sponged miR-590-5p to prevent it from binding the upstream molecule of STAT3. Targeting HOTAIR eliminated Docetaxel resistance in prostate cancer [85]. In OC, HOTAIR retarded the miR-206-mediated suppression of TBX3 levels and enhanced DDP drug resistance, providing a novel biomarker for OC treatment [86]. In another study, the HOTAIR level was upregulated in OC CSCs, which stimulated their colony-forming and spheroid-forming abilities. Interrupting the HOTAIR-EZH2 interaction and DNA methylation is likely to boost chemosensitivity and restrain cancer recurrence [87].

**Table 5:** HOTAIR enhances the self-renewal ability of cancer stem cells.

Cancer type	Expression	Related drugs	Target	Related genes or pathway	References
Lung cancer	upregulation	cisplatin	/	Klf4	[82]
Pancreatic cancer	upregulation	gemcitabine	/	/	[83]
Prostatic cancer	upregulation	docetaxel	miR-590-5p	STAT3	[85]
Ovarian cancer	upregulation	cisplatin	miR-206	TBX3	[86]
Ovarian cancer	upregulation	platinum/paclitaxel	/	/	[87]

Thus, several important signaling pathways have been shown to be involved in the self-renewal of CSCs, including the Bmi1, Notch, Wnt, and Hedgehog signaling pathways [88–91]. HOTAIR enhances the self-renewal ability of CSCs and plays a vital role in cancer chemosensitivity and recurrence.

### HOTAIR interferes with the DNA repair pathway

The DNA in every cell of the human body is suffered tens of thousands damage each day. DNA repair is a cellular response to DNA damage aiming to restore the DNA structure and function. DNA repair protects the genome from damage and mutation and is therefore important for cell survival [92]. Fortunately, cells contain a variety of DNA repair mechanisms, including: (1) nucleotide excision repair (NER), which removes large amounts of DNA adducts; (2) base excision repair (BER), which removes damaged bases; (3) mismatch repair (MMR), which recognizes base damage and incorporation errors; (4) cross-linking repair (ICLR), which removes interstrand cross-links; (5) nonhomologous end joining (NHEJ) and homologous recombination (HR), which repairs DNA backbone damage; (6) DNA damage response (DDR), which deals with multi-step complex DNA damage repair.

Qian et al. [93] revealed that overexpression of HOTAIR promoted the expression of DDR factors, including DNA-protein kinases, Ku protein (Ku70 and Ku80), and ATM. Alternatively, these signaling molecules in the double-strand break repair pathway can be blocked by EZH2. Another research group demonstrated that DNA damage induced HOTAIR expression in a p53-dependent manner, indicating that HOTAIR involved in complex DDR. Therefore, HOTAIR might mediate the resistance of oral squamous cells to doxorubicin and irradiation [68]. Similar results indicated that HOTAIR silencing sensitized BCa cells to radiation, induced DNA damage, suggesting that HOTAIR may be a novel theraputic target for BCa [94]. In addition, Gao et al. [95] reported that HOTAIR was positively correlated with the degree of DNA damage, which indicated that HOTAIR may be involved in the modulation of DNA damage induced by PAHs exposure. Gupta et al. [8] demonstrated that enhanced expression of HOTAIR induced genome-wide retargeting of PRC2, resulting in altered H3K27me3 gene expression, and increased cancer invasion and metastasis in a PRC2-dependent manner during BC progression. Similar results indicated that the HOTAIR level was associated with genome-wide reprogramming of PRC2 function and that HOTAIR upregulation could be a key factor in the progression of colorectal cancer (CRC) metastasis [96]. Recently, HOTAIR was found to affect chemotherapy resistance by interfering with DNA repair (Table 6). Liu et al. [50] reported that HOTAIR recruited EZH2 to transcriptionally inhibit the expression of p21^WAF1/CIP1^ by modifying H3K27me3, thereby triggering DDP resistance by inhibiting p21-dependent DNA repair suppression in NSCLC. Another study showed that HOTAIR modulated the activation of NF-κB by reducing the NF-κB inhibitor Iκ-Bα in OC. Furthermore, they demonstrated that HOTAIR played a key role in platinum chemotherapy resistance and cellular senescence by inducing prolonged NF-κB activation and expression of NF-κB target genes during DNA damage [97].

**Table 6:** HOTAIR participates in interfering with DNA repair pathway.

Cancer type	Expression	Related drugs	Target	Related genes or pathway	References
Oral cavity carcinoma	upregulation	doxorubicin	/	/	[68]
Lung cancer	upregulation	cisplatin	/	P21	[50]
Ovarian cancer	upregulation	platinum	/	NF-κB	[97]

Together, these studies demonstrate that HOTAIR participates in chemotherapy resistance by interfering with the DNA repair pathway.

### HOTAIR affects the drug efflux pump

Scientists had demonstrated that a major factor in the development of MDR in cancer cells was the reduction of drug accumulation. Approximately 40 years ago, Juliano et al. [98] discovered that P-gp mediates the active flow of chemotherapeutic drugs from cancer cells, resulting in pleiotropic drug cross-resistance.

A multitude of data has shown that HOTAIR causes chemotherapy resistance by modulating ABC transporters to affect the drug efflux pump (Table 7). In GC, the evidence demonstrated that the high expression of HOTAIR was associated with DDP resistance. HOTAIR could directly bind and inhibit the expression of miR-126, thereby activating the PI3K/AKT/MRP1 pathway [54]. A year later, similar results indicated that downregulation of HOTAIR might enhance the sensitivity of CML cells to imatinib through the PI3K/AKT/MRP1 pathway [34]. In HCC, Zhou et al. [99] reported that HOTAIR silencing impaired STAT3 activity and multi-drug resistant protein 1 (MDR1, ABCB1) expression and decreased chemotherapy resistance to DDP. Therefore, HOTAIR could become a new potential therapeutic target for the reversal of MDR in HCC. Subsequent studies showed that TGF-β1 was involved in a novel MDR mechanism by upregulating BCRP and P-gp through the SMAD4/HOTAIR/miR-145 pathway in HCC [100]. In NSCLC, an in-depth study revealed that si-HOTAIR enhanced the sensitivity of cells to DDP by inhibiting the expression levels of MRP1 and MDR1, as well as the Wnt signaling pathway [40].

**Table 7:** HOTAIR participates in affecting the drug efflux pump.

Cancer type	Expression	Related drugs	Target	Related genes or pathway	References
Gastric cancer	upregulation	cisplatin	miR-126	PI3K/AKT/MRP1	[54]
Leukemia	upregulation	platinum	/	PI3K/AKT/MRP1	[34]
Hepatocellular cancer	upregulation	cisplatin	/	MDR1	[99]
Hepatocellular cancer	upregulation	imatinib	miR-145	P-gp and BCRP	[100]
Lung cancer	upregulation	cisplatin	/	MRP1, MDR1 and Wnt	[40]

These results confirm that HOTAIR is involved in chemotherapy resistance by affecting the drug efflux pump in human tumors.

### HOTAIR influnces the TME

The TME refers to the close relationship between the occurrence, growth, and metastasis of tumors and the internal and external environment of tumor cells. It is a hypoxic and acidic environment, with high osmotic pressure, containing microbiota, inflammatory factors, tumor-associated macrophages, extracellular matrix and molecules, and cancer-associated fibroblasts.

Several studies have revealed that HOTAIR influences drug resistance in an altered TME (Table 8). In 2017, a peptide nucleic acid (PNA)-mediated approach was discovered to block and inhibit the activity of the HOTAIR-EZH2 interaction, which resensitized resistant OC to platinum. Mechanically, PNAs were conjugated to pH-low insertion peptide (pHLIP) to facilitate the delivery of anti-HOTAIR effects to an acidic (pH about 6) TME. The pHLIP-PNA complex suppressed the activity of HOTAIR and decreased tumor formation, suggesting a new approach for the treatment of solid tumor drug resistance [101].

**Table 8:** HOTAIR influnces tumor microenvironment.

Cancer type	Expression	Related drugs	Target	Related genes or pathway	References
Ovarian cancer	upregulation	platinum	/	/	[101]

Overall, these data indicate that HOTAIR influences chemoresistance in an altered TME.

## Therapeutic potential of HOTAIR in cancer

A large number of studies have shown the effect of HOTAIR on the occurrence, progression, and metastasis of multiple tumors. Therefore, treatment strategies based on HOTAIR, including RNA interference (RNAi), small molecule inhibitors and antisense oligonucleotides (ASOs), have attracted wide attention. Gupta et al. [8] adopted RNAi technology to target HOTAIR and create HOTAIR knockout. Similarly, Lennox et al. [102] found that both RNAi and ASOs effectively inhibited HOTAIR. In addition, Bhan et al. [103] invented a synthetic small interfering nucleotide DNA complementary to the HOTAIR transcript to suppress the expression of HOTAIR.

Moreover, there are some inhibitors that suppress HOTAIR function without altering its expression level. For instance, Li et al. [104] discovered a small molecule compound, AC1Q3QWB (AQB), functioning as a HOTAIR-EZH2 inhibitor to block PRC2 recruitment. One year later, Shi et al. [105] demonstrated that the combination of AQB and palbociclib led to a higher inhibition of growth and metastasis than that obtained using the single drug in glioma cells with high HOTAIR expression. Ozes et al. [101] revealed that overexpression of HOTAIR blocked the binding of HOTAIR to EZH2, thereby decreasing tumor invasion and increasing chemotherapy sensitivity in breast and ovarian cancer cells. Therefore, screening a larger library of natural and synthetic compounds to identify compounds that target the interaction between HOTAIR and PRC2 complexes could be an alternative strategy for targeting HOTAIR/ EZH2-dependent cancers.

The exact molecular mechanisms by which HOTAIR is involved in cancer progression are more intricate than originally thought and have not yet been applied in the clinic. More research is needed to further understand the role of HOTAIR in the pathogenesis of human cancers.

## Conclusions and prospects

Chemotherapy is a cancer treatment that involves the use of chemical drugs to prevent the proliferation, invasion, and metastasis of cancer cells and to finally kill them. It is a systemic treatment and together with surgery and radiation constitutes the three major cancer treatment types. However, tumor cells can develop resistance to chemotherapeutic drugs, resulting in a significant reduction in the chemotherapeutic effect of the drugs. Therefore, chemotherapy resistance is problem that needs to be urgently solved.

Recently, lncRNAs have been widely recognized as an important biological regulator of the tumor progression process. Furthermore, some lncRNAs, including HOTAIR, have been found to be involved in chemotherapy resistance [3, 32, 106, 107]. According to the literature, HOTAIR-induced MDR has been shown to increase drug resistance and decrease drug sensitivity in a variety of cancers. In this review, we explored the different mechanisms by which HOTAIR induces drug resistance in various tumors and showed that HOTAIR could confer resistance to multiple anticancer drugs, such as doxorubicin, ADM, imatinib, DDP, 5-FU, taxol, progesterone, TMZ, VP-16, gefitinib, PTX, DEX, sorafenib, mitomycin, trastuzumab, crizotinib, sunitinib, gemcitabine, docetaxel, platinum, PTX, and oxaliplatin. Furthermore, a series of miRNAs were involved in HOTAIR-mediated chemotherapy resistance, such as miR-34a, miR-106a-5p, miR-125, miR-126, miR-217, miR-17-5p, miR-519a-3p, miR-130a, miR-590-5p, miR-206, and miR-145. Moreover, the Wnt/β-catenin and PI3K/Akt signaling pathways were two important mediators of HOTAIR chemoresistance. In addition, we found that most studies used RNA-seq to screen lncRNAs with different expression patterns between resistant and non-resistant cell lines to explore the mechanisms of chemotherapy resistance, and targeting these differentially expressed lncRNAs might contribute to the elimination of chemotherapy resistance.

According to literature reports, we can see that the current research on the role of HOTAIR in tumor chemotherapy resistance is still in its infancy. There are still some challenges to be solved. First, studies of HOTAIR in cancer chemotherapy resistance are limited to a few classic drugs and a few cancer types. More drugs and cancer types need to be investigated. Second, HOTAIR polymorphisms and their relationship with chemotherapy response have not been evaluated in a large cohort of patients. Therefore, future studies should be performed to fill this research gap. Third, although many studies revealed that HOTAIR was associated with chemotherapy resistance, few studies investigated how drug resistance can be reduced by inhibiting HOTAIR expression in clinical practice. Further clinical studies may provide a new direction for exploring the mechanisms of HOTAIR-related drug resistance.

In conclusion, HOTAIR is closely involved in tumor development and associated with chemotherapy resistance. We summarized the various mechanisms by which HOTAIR leads to tumor resistance and conclude that HOTAIR may play an important role in cancer therapy in the future. Therefore, further exploration in this field could provide additional approaches to the treatment of tumor drug resistance.
